# Syndecan and integrin interactomes: large complexes in small spaces

**DOI:** 10.1016/j.sbi.2012.07.003

**Published:** 2012-10

**Authors:** James A Roper, Rosalind C Williamson, Mark D Bass

**Affiliations:** School of Biochemistry, University of Bristol, University Walk, Bristol BS8 1TD, United Kingdom

## Abstract

The syndecan family of transmembrane proteoglycans cooperate with integrins to regulate both early and late events in adhesion formation. The heparan sulphate chains substituted on to the syndecan ectodomains are capable of engaging ligands over great distance, while the protein core spans the plasma membrane and initiates cytoplasmic signals through a short cytoplasmic tail. These properties create a spatial paradox. The volume of the heparan sulphate chains greatly exceeds that of the integrins with which it cooperates, while the short cytodomain must bind to multiple cytoplasmic factors, despite being long enough to bind only one or two. In this review we consider the structural rearrangements that a cell undertakes to overcome spatial restrictions and compare the interactomes of syndecans and integrins to gain insight into the composition of adhesions and how they are regulated over time.

## Highlights

► Syndecans are cleared from adhesion sites to allow integrin engagement. ► Literature-curated interactomes differ between syndecan-1 and syndecan-4. ► Overlap between interactomes of syndecan or integrin describe adhesion formation. ► Switching of syndecan signalling by phosphorylation.

**Current Opinion in Structural Biology** 2012, **22**:583–590

This review comes from a themed issue on **Carbohydrates and glycoconjugates**

Edited by **Alisdair B Boraston** and **David Fernig**

For a complete overview see the Issue and the Editorial

Available online 26th July 2012

0959-440X/$ – see front matter, © 2012 Elsevier Ltd. All rights reserved.

**http://dx.doi.org/10.1016/j.sbi.2012.07.003**

## Introduction

Heparan sulphate proteoglycans (HSPGs) are among the most abundant cell-surface receptors and engage a wide range of extracellular ligands, such as extracellular matrix (ECM) molecules, growth factors and chemokines. HSPGs can be divided into the syndecans and glypicans, both comprising a protein core substituted with 3–5 heparan sulphate (HS) chains, and also chondroitin sulphate in some cases. It is through the HS chains that HSPGs engage extracellular ligands, and the length and flexibility of HS allows the capture of distant or dilute ligands, making HSPGs ideal early sensors of changes in the extracellular environment. Although syndecans and glypicans are each substituted with HS, and therefore capture similar ligands, there is a major difference between the two families. Glypicans are anchored to the membrane by a GPI linkage, and therefore are limited to organising ligands outside the cell. By contrast, syndecans include a transmembrane domain, a short cytoplasmic domain, and form constitutive homodimers, which means that they can organise formation of multimolecular complexes on the cytoplasmic face of the plasma membrane, in response to extracellular ligand binding. It is the effect of syndecans on multimolecular complex assembly that we shall consider in this review.

## Syndecan-integrin synergy

Syndecans cooperate with a number of integrins to regulate adhesion to a variety of ECM ligands and, surprisingly, the mechanisms are quite diverse. Syndecan-4 cooperates with α_5_β_1_-integrin [1] and α_v_β_3_-integrin [2] to promote focal adhesion assembly around pre-existing integrin clusters on fibronectin. By contrast, syndecan-1 activates α_v_β_3_-integrin and α_v_β_5_-integrin by forming a ternary complex with the insulin-like growth factor receptor and regulating activation of the integrins by talin [3^••^]. In yet another mechanism, syndecan-1 is absolutely necessary for α_2_β_1_-integrin-mediated interaction with collagen, both in 2D and 3D, but this time due to generation of tension in the actin cytoskeleton [4]. Furthermore, syndecan-4 promotes collagen contraction in 3D, but has no effect on α_2_β_1_-integrin in 2D [5^••^]. The range of mechanisms reflect the fact that integrin-syndecan crosstalk depends on the interplay of two multi-component signalling complexes, rather than a single switch.

**Figure 1:**
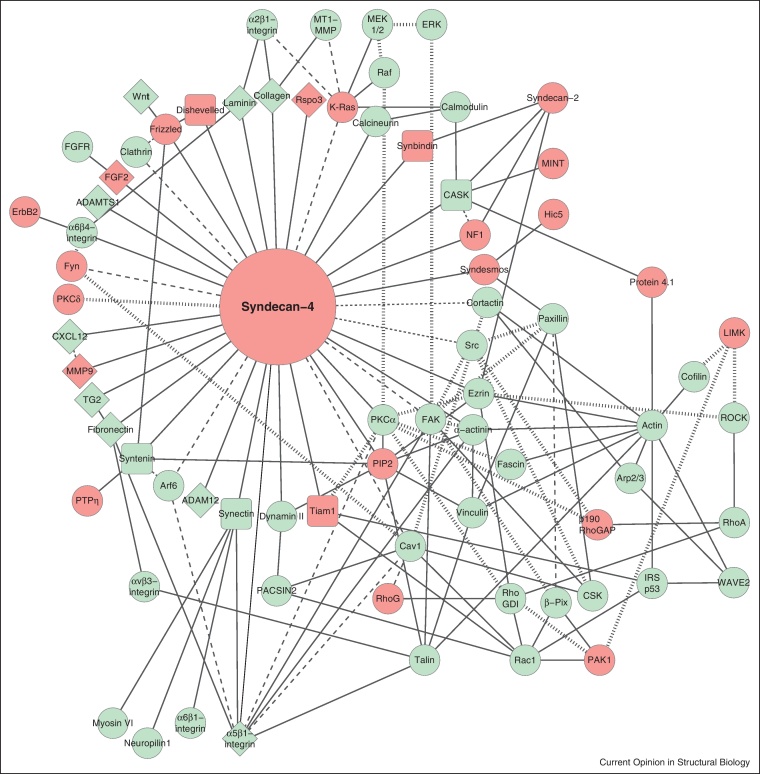
Literature-curated syndecan-4 interactome. Interactions are classified as direct (solid line), indirect (dotted line) or phosphorylation events (grey lines). Proteins can be classified as those identified in proteomic analyses of integrin complexes (green) and those excluded (red). Proteins involved in extracellular interactions are shown as diamonds, those that interact through a PDZ domain as squares. The complete reference list for interactions can be found in supplementary Table S1.

**Figure 2:**
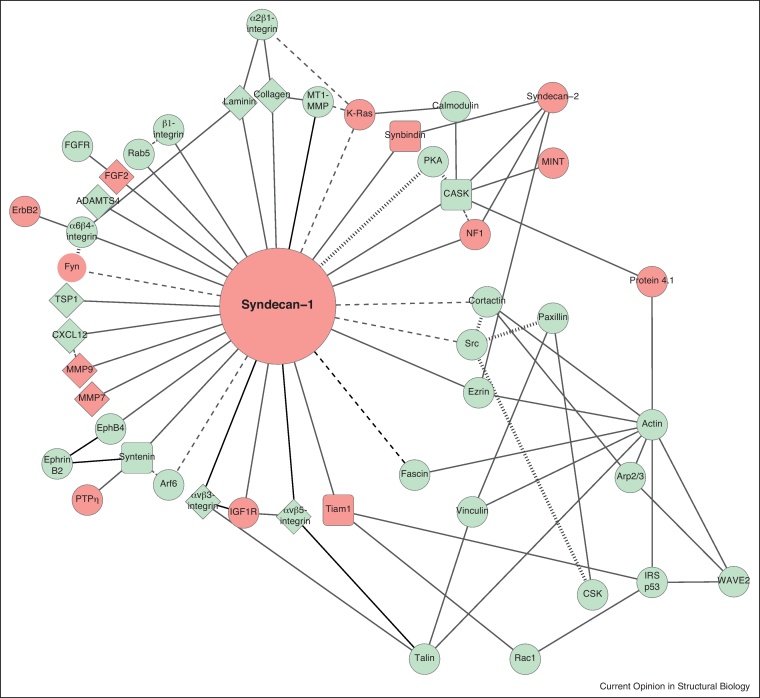
Literature-curated syndecan-1 interactome. Interactions are classified as direct (solid line), indirect (dotted line) or phosphorylation events (grey lines). Proteins can be classified as those identified in proteomic analyses of integrin complexes (green) and those excluded (red). Proteins involved in extracellular interactions are shown as diamonds, those that interact through a PDZ domain as squares. The complete reference list for interactions can be found in supplementary Table S2.

Proteomic analyses of integrin complexes have allowed the actual composition of focal adhesions to be catalogued [6–8]. Each study identified over 400 focal adhesion components, of which some were conserved, but others varied between the integrin studied. Proteomic analyses have been complemented by generation of literature-curated integrin interactomes that have 156 components [9]. Similar studies have not been conducted for the syndecans, and an analysis of proteins that interact with syndecans by mass spectrometry is now overdue. We have compiled literature-curated interactomes of syndecan-1 and syndecan-4 and find that the syndecan-4 interactome is considerably more complex than that of syndecan-1 (Figures 1 and 2 and Tables S1 and 2). The additional complexity is almost entirely due to the formation of a syndecan-4/PI(4,5)P_2_/PKCα ternary complex that mediates many syndecan-4-dependent signalling responses [10,11]. Significantly, there is only partial overlap (Figures 1 and 2, green nodes) between the literature-curated syndecan interactomes and the experimentally determined integrin interactomes, indicating that the receptors are not constitutively associated. Integrins are found on both syndecan interactomes, and involve both cytoplasmic and extracellular interactions. Cytoplasmic interactions include binding of β_1_-integrins to the syndecan-1 cytodomain [12] and binding of α_6_β_4_-integrin to the C-terminal motif of all syndecans [13]. Extracellular interactions with integrins include binding of α_v_β_3_-integrins and α_v_β_5_-integrins to the synstatin peptide of the syndecan-1 ectodomain [14] and binding of β_1_-integrins to the NXIP motif of syndecan-4 ectodomains [15]. The stark difference between which integrin/syndecan complexes are biochemically possible versus which are actually observed in the cell makes it crucial that we begin to understand the spatial relationship between syndecans and integrins.

## Exclusion of syndecans from focal adhesions by spatial constraints

**Figure 3:**
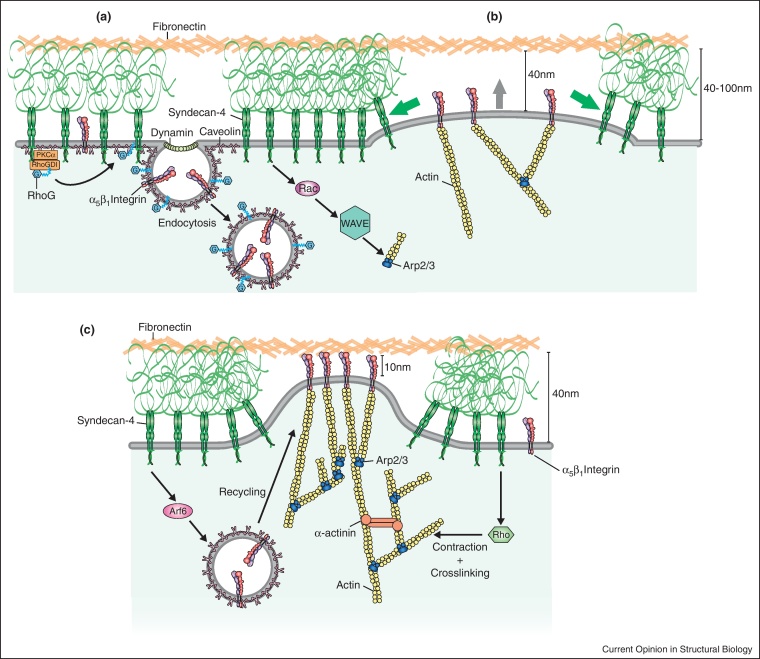
Spatial rearrangement of syndecan and integrin during adhesion formation. **(a)** Syndecan-4 detects fibronectin that is greater than 40 nm from the plasma membrane and triggers RhoG/caveolin-dependent endocytosis of integrin and Rac1-dependent polymerisation of branched actin filaments. **(b)** Actin polymerisation causes local membrane protrusion that causes lateral movement of syndecan-4 due to spatial constraints as the gap between membrane and matrix decreases to 10–40 nm. **(c)** Once the plasma membrane is within 10 nm of the ECM, integrin engages fibronectin, forming a nouveau adhesion. Integrin is recycled through an Arf6-dependent pathway. Peripheral syndecan-4 activates RhoA to cause contraction and bundling of the actin cytoskeleton.

It is extremely noticeable that syndecans were not identified in any of the proteomic analyses of integrin complexes. Mathematical models of the spatial constraints applied to a focal adhesion have indicated why this might be the case [16^••^]. The HS chains of syndecans have a potential reach of 500 nm [17], which is ideal for capture of distant ligands, but also provides a physical barrier to close association between plasma membrane and ligand. The flexible HS chains can be compressed, but cryo-electron microscopic studies have revealed that a 40-nm thick coat of glycocalyx still remains [18]. Integrins by contrast have a maximum reach of 17 nm [19], and the distance between plasma membrane and ligand in a focal adhesion is closer to 10 nm (Figure 3). These spatial constraints would, by necessity, exclude syndecans from the mature integrin complexes analysed by mass spectrometry. It is proposed, therefore, that syndecans make the initial contacts with the ECM, but are then moved laterally by localised actin polymerisation to allow integrin engagement [16^••^].

What this means is that direct links between syndecan and integrin should prime and position the integrin for binding to the ECM. Active, but unligated, integrins cluster at the polymerising tips of actin within a lamella [20] and actin polymerisation supplies the energy for localised membrane protrusion that causes the initial integrin contact with the ECM [16^••^]. The Rac1 GEF, Tiam1, has been reported to bind to the C-termini of syndecan-1, syndecan-2 and syndecan-4 [21,22^•^], while regulation of Rac1 by syndecan-4 has been well documented, both *in vitro* and *in vivo* [1,23]. Thus, the Tiam1/Rac1/WAVE2 link from syndecans to the actin nucleator, Arp2/3, may well provide the mechanism for nucleation of nouveau integrin adhesions. A second key finding has been that engagement of syndecan-4 triggers endocytosis and recycling of α_5_β_1_-integrin to allow formation of new focal complexes [24^••^,25]. The endocytic event is regulated by a PKCα/RhoGDI/RhoG complex, and so is exclusive to syndecan-4 [26]. It is noticeable that neither Tiam1, RhoG, nor the RhoA inhibitor, p190RhoGAP were detected in the integrin complexes. Activation of Rac1, RhoG and p190RhoGAP-mediated inhibition of RhoA [27] are each understood to be early responses to syndecan-4 engagement that drive membrane protrusion and focal complex formation. When combined with the demonstration of integrin activation by syndecan-1, it becomes apparent that syndecans act as early ECM sensors that initiate focal adhesion formation. This is not to dispute the well-documented role of syndecan-4 in focal adhesion maturation through processes such as RhoA activation [28], but clearly such cooperation must involve the syndecan surrounding, rather than within the focal adhesion itself.

## Competition for space around the syndecan cytoplasmic domains

**Figure 4:**
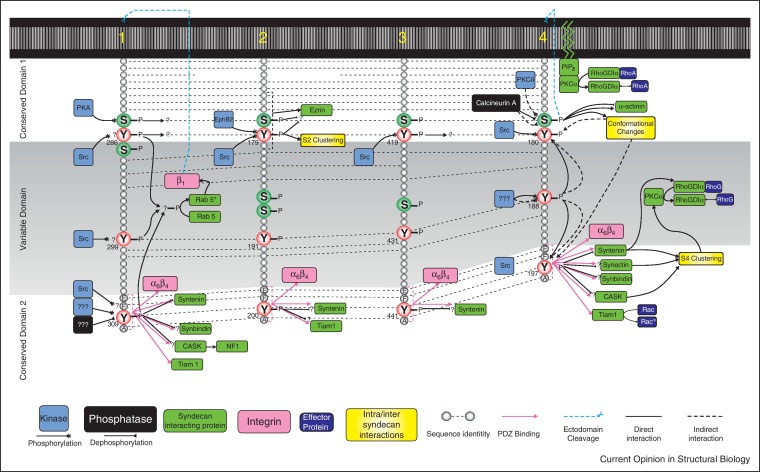
Phosphorylation sites and subsequent protein interactions of the syndecan cytoplasmic domains. Serine and tyrosine residue phosphorylation by kinases and phosphatases are depicted for each syndecan, together with the consequential binding of effector proteins. Interactions are classified as direct (solid lines) and indirect (dotted lines) with those that bind via a PDZ domain shown with pink lines. Residue numbers correspond to the human sequences; asterisks indicate active protein forms. Although some of the interactions have not been empirically determined, those that have been reported are tabulated in supplementary Table S3.

The syndecan cytoplasmic domains are divided into conserved or variable regions according to the sequence homology with other members of the family. Two conserved domains (C1 and C2) permit binding of a host of proteins that may be common to all syndecans whilst a variable domain (V) allows interaction with proteins unique to each syndecan. Interactions of the conserved and variable motifs are necessary for signal transduction, as syndecans lack inherent enzymatic activity. The large number of putative binding partners, combined with the short length of the syndecan cytodomains (28–34 residues) means that there is a great deal of competition for space between binding partners. Phosphorylation of key residues regulates the binding of some of these partners and provides a mechanism by which clustering and signal transduction by syndecans can be regulated. There are several conserved tyrosine and serine residues common to the cytoplasmic regions of all four syndecans that may offer a conserved method of regulating syndecan signalling. These include the serine–tyrosine residue pair in the C1 domain, and a single tyrosine in the C2 domain (Figure 4 and Table S3).

The C2 domain comprises a C-terminal EFYA motif that is common to all syndecans and constitutes a PDZ domain-binding site that mediates protein–protein interactions with a number of binding partners (Figures 1 and 2, square nodes). The importance of the EFYA motif is apparent, as deletion of the motif blocks syndecan-4-dependent cell migration [29]. Proteins that bind to the PDZ-binding site often participate in signalling downstream of the syndecan (e.g. Tiam1 acting as a Rac1 GEF, CASK influencing calcium release), are involved in the regulation of trafficking (e.g. synectin, synbindin), and recycling (e.g. syntenin in concert with Arf6) or act as scaffold proteins to nucleate larger complexes (e.g. CASK). Although the C2 domain is not directly involved in syndecan dimerisation, NMR studies demonstrate that the orientation of EFYA motifs of dimeric syndecans is ideal for both simultaneous canonical and non-canonical interactions with dimeric syntenin complexes [30]. The large number of binding partners necessitates some form of specificity regulation. Binding of syndecan-1 to the second PDZ domain of syntenin is inhibited by phosphorylation of tyrosine-309 within the syndecan C2 domain [31^•^]. The PDZ domain of syntenin is well adapted to the syndecan EFYA motif, but too narrow to accommodate the phosphate group, and an unfavourable interaction between the phosphate and an aspartate residue of syntenin would further discourage binding. On the basis of sequence similarity, a similar mechanism would be predicted to occur in syndecan-4. The ubiquitously expressed Rac1 GEF Tiam1 also binds to the EFYA motif of syndecans via its PDZ domain [21,32]. Phosphorylation of tyrosine-309 of syndecan-1, which disrupts syntenin binding, has no effect on Tiam1 binding [22^•^]. The difference is observed because the side-chain interaction of tyrosine-309 with syntenin does not occur in Tiam1. Crucially, syndecan-1 phosphorylation diminishes during adhesion formation [31^•^] allowing a switch from Tiam1-mediated membrane protrusion to syntenin-mediated syndecan redistribution, as the adhesion forms. Therefore, it would seem that phosphorylation of the syndecan C2 domain can provide a specificity switch that goes some way to resolving the overcrowding issue. However, the fact that there are six PDZ-domain proteins plus α_6_β_4_-integrin competing to bind to the EFYA means that there must be additional factors, be they regulatory modifications, such as phosphorylation or spatial constraints within the cell.

## The regulatory serine residue of the syndecan cytoplasmic domain

As we have already noted, the signalling properties of syndecan-4 rely on formation of a complex between PI(4,5)P_2_, PKCα and the syndecan-4 variable domain. The variable domain in syndecan-4 forms a secondary twisted clamp structure not observed in other syndecans, where oligomerisation occurs due to interactions between transmembrane domains of each subunit. Phosphorylation of serine-179 in the C1 region of syndecan-4 by PKCδ negatively regulates PKCα activation and formation of the twisted clamp [10,33]. It has been suggested that the major substrate for PKCα in these circumstances may be RhoGDIα whose phosphorylation liberates RhoG and RhoA, resulting in focal adhesion initiation and maturation, respectively [24^••^,26,28]. Although phosphorylation of serine-179 has a negative effect on PKCα-binding, it may favour binding of α-actinin [34], providing a second example of a phosphorylation-dependent specificity switch. However, the finding is disputed by Choi *et al.*, who suggest that phosphorylation of this serine reduces α-actinin binding [35]. However, this discrepancy may be due to differences in cell types used and hints at possible divergent roles for α-actinin coupling to syndecan-4 in different circumstances.

Intriguingly, phosphorylation of serine-179 has also been reported to be necessary for shedding of the syndecan-4 ectodomain that was in turn necessary for progression of cells through mitosis [36]. The fact that phosphorylation of the same residue has been linked to regulation of cell spreading through RhoG and Rac1, and regulation of mitosis through shedding provides yet another example that the context of the individual interactions of the syndecan-4 cytodomain are as important as the interactions themselves. The equivalent residue of syndecan-1 (serine-285) is phosphorylated by PKA in response to stimulation of cells with TGFβ [37]. Although binding interactions affected by this event have not been identified, phosphorylation increases the surface expression of syndecan-1, and, therefore, appears to fulfil a very different role to phosphorylation of serine-179 in syndecan-4. Nevertheless, the interaction of syndecans with several key signalling molecules is critically regulated by a single phosphorylation site at the C1/V junction that may dictate the spatial constraints of any potential binding proteins.

Further regulation of serine-179 phosphorylation comes in the form of the calmodulin dependant protein phosphatase, calcineurin, which preferentially binds to syndecan-4 when serine-179 is dephosphorylated [38]. Calcineurin possesses intrinsic phosphatase activity and thus can dephosphorylate serine-179 and regulate its own binding to syndecan-4. A further regulatory mechanism that has been poorly explored is the tyrosine residue that immediately follows the equivalent of serine-179 in all syndecans. Phosphorylation of this tyrosine by EphB2 induces syndecan-2 clustering in rat hippocampal neurons and is necessary for spine formation [39]. The tyrosine residues of the syndecan-1 cytodomain are necessary for agonist-induced shedding of the ectodomain. Surprisingly, shedding is not due to tyrosine phosphorylation, and instead depends on release of bound, inactive Rab5 [12]. Nevertheless, one would expect phosphorylation of the Rab5-binding site to have some effect when it occurs. Certainly, the possibility of phosphorylation of the tyrosine residue at the C1/V junction in other syndecans has yet to be explored, and given the proximity to the critical serine-179, such an investigation is now due.

## Conclusions

In this review, we have discussed numerous examples of syndecans responding to extracellular and cytosolic factors by recruiting a range of signalling molecules to the short cytoplasmic tail, behaviour that creates a great deal of competition for space around both extracellular and cytoplasmic domains. It is clear that cooperation between syndecans and integrins is required for focal adhesion formation despite the difference in the reach of the two receptors — a paradox that demands spatial segregation of the receptors and is demonstrated by the absence of syndecans from global analyses of integrin adhesions. As with many signalling networks, the key question now is how the alternative binding interactions are managed. We can propose several layers of regulation:

(1) Phosphorylation or dephosphorylation of specific residues favours binding of specific proteins.
(2) Recruitment of an initial binding partner may sterically hinder or stabilise recruitment of further partners.
(3) Conformational rearrangements of syndecan cytoplasmic regions occur due to binding of proteins or phosphorylation of residues.
(4) Syndecans included in nascent adhesions or excluded from mature adhesions will be associated with different complements of other transmembrane receptors that will generate their own microenvironment.

Such factors are necessary to confer specificity in what would otherwise be an extremely congested environment, and an unbiased, holistic approach to determining the composition of such complexes is now essential. The second key challenge is understanding the potential redundancy between syndecans. In some cases syndecan-1 and syndecan-4 appear capable of eliciting similar responses, but due to the unique twisted clamp configuration of the syndecan-4 cytodomain, the mechanisms are different. Therefore, understanding the spatial organisation at both the submolecular and whole focal adhesion level is now crucial.

## References and recommended reading

Papers of particular interest, published within the period of review, have been highlighted as:

• of special interest
•• of outstanding interest
