# Supplementary material for: Syndecan and integrin interactomes: large complexes in small spaces
Source: Curr Opin Struct Biol. 2012 Oct;22(5):583–90. doi: 10.1016/j.sbi.2012.07.003 (PMC3712168; doi:10.1016/j.sbi.2012.07.003)
Supplement: Supplementary file 1 [file mmc1.docx]

| **Partner A** | **Type** | **Partner B** | **Reference** |
| --- | --- | --- | --- |
| α-actinin | D | Actin | Ebashi, Ebashi & Maruyama (1964) Nature 203, 645-6 |
| α-actinin | D | Vinculin | Kelly, Taylor, Bakolitsa, Bobkov, Bankston, Liddington & Taylor (2006) J. Mol. Biol. 357, 562-73 |
| α5β1-integrin | D | α-actinin | Otey, Pavalko & Burridge (1990) J. Cell Biol. 111, 721-9 |
| α5β1-integrin | I | Arf6 | Brooks, Williamson, Bass (2012) Small GTPases |
| α5β1-integrin | I | Caveolin | Del Pozo, Balasubramanian, Alderson, Kiosses, Grande-Garcia, Anderson & Schwartz (2005) Nat. Cell Biol. 7, 901-8 |
| α5β1-integrin | D | FAK | Schaller, Otey, Hildebrand & Parsons (1995) J. Cell Biol. 130, 1181-7 |
| α5β1-integrin | I | PKCa | Ng, Shima, Squire, Bastiaens, Gschmeissner, Humphries & Parker (1999) Embo J. 18, 3909-23 |
| α5β1-integrin | D | Synectin | Valdembri, Caswell, Anderson, Schwarz, Konig, Astania, Caccavari, Norman, Humphries, Bussolino & Serini (2009) 7, e1000025 |
| α5β1-integrin | D | Talin | Tadokoro, Shattil, Eto, Tai, Liddington, de Pereda, Ginsberg & Calderwood (2003) Science 302, 103-6 |
| α6β1-integrin | D | Synectin | Mourabit, Poinat, Koster, Sondermann, Wixler, Wegener, Laplantine, Geerts, Georges-Labouesse, Sonnenberg & Aumailley (2002) Matrix Biol. 21, 207-14 |
| Actin | D | IRSp53 | Takenawa & Suetsugu (2007) Nat.Rev.Mol.Cell Biol. 8, 37-48 |
| Actin | D | WAVE2 | Suetsugu, Miki, Yamaguchi, Obinata & Takenawa (2001) J. Cell Sci. 114, 4533-42 |
| Arp2/3 | D | Actin | Blanchoin, Amann, Higgs, Marchand, Kaiser & Pollard (2000) Nature 404, 1007-11 |
| Arp2/3 | D | Cortactin | Uruno, Liu, Zhang, Fan, Egile, Li, Mueller & Zhan (2001) Nature Cell Biol. 3, 259-66 |
| αvβ3-integrin | D | Talin | Wegener, Partridge, Han, Pickford, Liddington, Ginsberg & Campbell (2007) Cell 128, 171-82 |
| β-pix | D | PAK1 | Brown, West & Turner (2002) Mol. Biol. Cell 13, 1550-65 |
| β-pix | I | Paxillin | Brown, West & Turner (2002) Mol. Biol. Cell 13, 1550-65 |
| β-pix | D | Rac1 | ten Klooster, Jaffer, Chernoff & Hordijk (2006) J. Cell Biol. 172, 759-69 |
| Calcineurin | D | Calmodulin | Finsen, Lunde, Sjaastad, Ostil, Lyngra, Jarstadmarken, Hasic, Nygard, Wilcox-Adelman, Goetinck, Lyberg, Skrbic, Florholmen, Tonnessen, Louch, Djurovic, Carlson & Christensen (2011) Plos One, 6, e28302 |
| Calmodulin | D | K-Ras | Villalonga, Lopez-Alcala, Bosch, Chiloeches, Rocamora, Gil, Marais, Marshall, Bachs & Agell (2001) Mol. Cell Biol. 21, 7345-54 |
| CASK | D | Calmodulin | Borg, Lopez-Figueroa, Taddeo-Borg, Kroon, Turner, Watson & Margolis (1999) 19, 1307-16 |
| CASK | D | MINT1 | Borg, Lopez-Figueroa, Taddeo-Borg, Kroon, Turner, Watson & Margolis (1999) 19, 1307-16 |
| CASK | I | NF1 | Hsueh, Roberts, Volta, Sheng & Roberts (2001) J. Neuroscience 21, 3764-70 |
| CASK | D | Protein 4.1 | Cohen, Woods, Maratia, Walther, Chishti & Anderson (1998) J. Cell Biol. 13, 129-38 |
| Caveolin | D | CSK | Cao, Courchesne & Mastick (2002) J. Biol.Chem. 277, 8771-4 |
| Caveolin | D | PACSIN2 | Hansen, Howard & Nichols (2011) J. Cell Sci. 124, 2777-85 |
| Caveolin | D | Rac1 | Nethe, Anthony, Fernandez-Borja, Dee, Geerts, Hensbergen, Deelder, Schmidt & Hordijk (2010) J. Cell Sci. 123, 1948-58 |
| Cofilin | D | Actin | Ghosh, Song, Mouneimne, Sidani, Lawrence & Condeelis (2004) Science 304, 743-6 |
| Collagen | D | α2β1-integrin | Vuoriluoto, Hognas, Meller, Lehti & Ivaska (2011) Matrix Biol. 30, 207-17 |
| Cortactin | D | Actin | Uruno, Liu, Zhang, Fan, Egile, Li, Mueller & Zhan (2001) Nature Cell Biol. 3, 259-66 |
| CSK | D | FAK | Sabe, Hata, Osada, Nakagawa & Hanafusa (1994) Proc. Natl. Acad. Sci. USA 91, 3984-8 |
| CSK | P | Src | Cao, Courchesne & Mastick (2002) J. Biol.Chem. 277, 8771-4 |
| CXCL12 | I | MMP9 | Brule, Charnaux, Sutton, Ledoux, Chagneau, Saffar & Gattegno (2006) Glycobiology 16, 488-501 |
| Dynamin II | D | PACSIN2 | Rao, Ma, Vahedi-Faridi, Sundborger, Pechstein, Puchkov, Luo, Shupliakov, Saenger & Hauke (2010) Proc. Natl. Acad. Sci. USA 107,8213-8 |
| Dynamin II | D | PI(4,5)P2 | Lin & Gilman (1996) J. Biol. Chem. 271, 27979-82 |
| Ezrin | D | Actin | Algrain, Turunen, Vaheri, Louvard & Arpin (1993) J. Cell Biol. 120, 129-139 |
| Ezrin | D | PI(4,5)P2 | Hirao, Sato, Kondo, Yonemura, Monden, Sasaki, Takai, Tsukita & Tsukita (1996) J. Cell Biol. 135, 37-51 |
| Ezrin | D | RhoGDI | Hirao, Sato, Kondo, Yonemura, Monden, Sasaki, Takai & Tsukita (1996) J. Cell Biol. 135, 37-51 |
| FAK | P | α-actinin | Izaguirre, Aguirre, Hu, Lee, Schlaepfer, Aneskievich & Halmovich (2001) J. Biol. Chem. 276, 28676-85 |
| FAK | P | β-pix | Chang, Lemmon, Park & Romer (2007) Mol. Biol. Cell 18, 253-264 |
| FAK | P | ERK | Hunger-Glaser, Salazar, Sinnett-Smith &Rozengurt (2003) J. Biol. Chem. 278, 22631-43 |
| FAK | P | Paxillin | Richardson & Parsons (1996) Nature 380, 538-40 |
| Fascin | D | Actin | Adams, Clelland, Collett, Matsumura, Yamashiro & Zhang (1999) Mol. Biol. Cell 10, 4177–90 |
| FGF2 | D | FGFR | Kuriyama & Mayor (2009) Development 136, 575-84 |
| Fibronectin | D | α5β1-integrin | Danen, Aota, van Kraats, Yamada, Ruiter & van Muijen (1995) J. Biol. Chem. 270, 21612-8 |
| Fibronectin | D | αvβ3-integrin | Huveneers, Truong, Fassler, Sonnenberg & Danen (2008) J. Cell Sci. 121, 2452-62 |
| Fibronectin | D | TG2 | Scarpellini, Germack, Lortat-Jacob, Muramatsu, Billet, Johnson & Verderio (2009) J. Biol. Chem. 284, 18411-23 |
| Frizzled | I | Clathrin | Ohkawara, Glinka & Niehrs (2011) Dev. Cell 20, 303-14 |
| Frizzled | D | Dishevelled | Munoz, Moreno, Oliva, Orbenes & Larrain (2006) Nat. Cell Biol. 8, 492-500 |
| Frizzled | D | Syntenin | Luyten, Mortier, Van Campenhout, Taelman, Degeest, Wuytens, Lambaerts, David, Bellefroid & Zimmermann (2008) Mol. Biol. Cell 19, 1594-604 |
| K-Ras | I | α2β1-integrin | Vuoriluoto, Hognas, Meller, Lehti & Ivaska (2011) Matrix Biol. 30, 207-17 |
| K-Ras | I | MT1-MMP | Vuoriluoto, Hognas, Meller, Lehti & Ivaska (2011) Matrix Biol. 30, 207-17 |
| K-Ras | D | Raf | Telci, Wang, Li, Verderio, Humphries, Baccarini, Basaga & Griffin (2008) J. Biol. Chem. 283, 20937-47 |
| Laminin | D | α2β1-integrin | Hozumi, Suzuki, Nielsen, Nomizu & Yamada (2006) J. Biol. Chem. 281, 32929-40 |
| LIMK | P | Cofilin | Maekawa, Ishizaki, Boku, Watanabe, Fujita, Iwamatsu, Obinata, Ohashi, Mizuno & Narumiya (1999) Science 285, 895-8 |
| MEK1/2 | P | ERK | Huang, Jacobson, Scaller (2004) J. Cell Sci. 117, 4619-28 |
| MT1-MMP | D | Collagen | Vuoriluoto, Hognas, Meller, Lehti & Ivaska (2011) Matrix Biol. 30, 207-17 |
| Neuropilin1 | D | Synectin | Valdembri, Caswell, Anderson, Schwarz, Konig, Astania, Caccavari, Norman, Humphries, Bussolino & Serini (2009) 7, e1000025 |
| p190RhoGAP | D | RhoA | Bass, Morgan, Roach, Settleman, Goryachev & Humphries (2008) J. Cell Biol. 181, 1013-26 |
| PACSIN2 | D | Rac1 | de Kreuk, Nethe, Fernandez-Borja, Anthony, Hensbergen, Deelder, Plomann, Hordijk (2011) J. Cell Sci. 124, 2375-88 |
| PAK1 | P | LIMK | Edwards, Sanders, Bokoch & Gill (1999) Nat. Cell Biol. 1, 253-9 |
| PAK1 | P | RhoGDI | DerMardirossian, Schnelzer & Bokoch (2004) Mol. Cell 15, 117-27 |
| Paxillin | D | CSK | Turner (2000) Nat. Cell Biol. 2, E231-6 |
| PI(4,5)P2 | D | α-actinin | Fukami, Furuhashi, Inagaki, Endo, Hatano & Takenawa (1992) Nature 359, 150-2 |
| PI(4,5)P2 | D | PKCα | Koo, Jung, Shin, Han, Mortier, Zimmermann, Whiteford, Couchman, Oh & Lee (2006) J. Mol. Biol. 355, 651-63 |
| PI(4,5)P2 | D | Talin | Martel, Racaud-Sultan, Dupe, Marie, Paulhe, Galmiche, Block & Albiges-Rizo (2001) J. Biol. Chem. 276, 21217-27 |
| PI(4,5)P2 | D | Vinculin | Gilmore & Burridge (1996) Nature 381, 531-5 |
| PKCα | P | Ezrin | Ng, Parsons, Hughes, Monypenny, Zicha, Gautreau, Arpin, Gschmeissner, Verveer, Bastiens & Parker (2001) Embo J. 20, 2723-41 |
| PKCα | P | Fascin | Adams, Clelland, Collett, Matsumura, Yamashiro & Zhang (1999) Mol. Biol. Cell 10, 4177–90 |
| PKCα | P | p190RhoGAP | Bass, Morgan, Roach, Settleman, Goryachev & Humphries (2008) J. Cell Biol. 181, 1013-26 |
| PKCα | P | Raf | Carroll & May (1994) J. Biol. Chem. 269, 1249-56 |
| PKCα | P | RhoGDI | Dovas, Choi, Yoneda, Multhaupt, Kwon, Kang, Oh & Couchman (2010) J. Biol. Chem. 285, 23296-308. Elfenbein, Rhodes, Meller, Schwartz, Matsuda & Simons (2009) J. Cell Biol. 186, 75-83 |
| PKCα | P | Tiam1 | Fleming, Cassondra & Exton (1998) FEBS Lett. 429, 229-33 |
| Protein 4.1 | D | F-actin | Biederer & Sudhof (2001) J. Biol. Chem. 276, 47869-76 |
| Rac1 | D | IRSp53 | Takenawa & Suetsugu (2007) Nat.Rev.Mol.Cell Biol. 8, 37-48 |
| Rac1 | D | PAK1 | Bokoch (2003) Annu. Rev. Biochem. 72, 743-81 |
| Raf | P | MEK1/2 | Huang, Jacobson, Scaller (2004) J. Cell Sci. 117, 4619-28 |
| RhoA | D | ROCK | Dovas, Yoneda & Couchman (2006) J. Cell Sci. 119, 2837-46 |
| RhoG | I | Caveolin | Prieto-Sanchez, Berenjeno & Bustelo (2006) Oncogene 25, 2961-73 |
| RhoGDI | D | Rac1 | Del Pozo, Kiosses, Alderson, Meller, Hahn & Schwartz (2002) Nat. Cell Biol. 4, 232-9 |
| RhoGDI | D | RhoA | Dovas, Choi, Yoneda, Multhaupt, Kwon, Kang, Oh & Couchman (2010) J. Biol. Chem. 285, 23296-308. Elfenbein, Rhodes, Meller, Schwartz, Matsuda & Simons (2009) J. Cell Biol. 186, 75-83 |
| RhoGDI | D | RhoG | Elfenbein, Rhodes, Meller, Schwartz, Matsuda & Simons (2009) J. Cell Biol. 186, 75-83 |
| ROCK | P | Ezrin | Matsui, Maeda, Doi, Yonemura, Amano, Kaibuchi, Tsukita & Tsukita (1998) J. Cell Biol. 140, 647-57 |
| ROCK | P | LIMK | Maekawa, Ishizaki, Boku, Watanabe, Fujita, Iwamatsu, Obinata, Ohashi, Mizuno & Narumiya (1999) Science 285, 895-8 |
| Src | P | Cortactin | Kinnunen, Kaksonen, Saarinen, Kalkkinen, Peng & Rauvala (1998) J. Biol. Chem. 273, 10702-8 |
| Src | P | FAK | Calalb, Polte & Hanks (1995) Mol. Cell. Biol. 15, 954-63 |
| Src | P | p190RhoGAP | Arthur, Petch & Burridge (2000) Curr. Biol. 10, 719-22 |
| Src | P | Paxillin | Turner (2000) Nat. Cell Biol. 2, E231-6 |
| Syndecan-2 | D | CASK | Cohen, Woods, Maratia, Walther, Chishti & Anderson (1998) J. Cell Biol. 13, 129-38 |
| Syndecan-2 | D | Ezrin | Granes, Berndt, Roy, Mangeat, Reina & Vilaro (2003) FEBS Lett. 547, 212-6 |
| Syndecan-2 | D | NF1 | Hsueh, Roberts, Volta, Sheng & Roberts (2001) J. Neuroscience 21, 3764-70 |
| Syndecan-2 | D | NF1 | Hsueh, Roberts, Volta, Sheng & Roberts (2001) J. Neuroscience 21, 3764-70 |
| Syndecan-2 | D | Synbindin | Ethell, Hagihara, Miura, Irie & Yamaguchi (2000) J. Cell Biol. 151, 53-67 |
| Syndecan-4 | D | α-actinin | Greene, Tumova, Couchman & Woods (2003) J. Biol. Chem 278, 7617-23 |
| Syndecan-4 | D | ADAM12 | Iba, Albrechtsen, Gilpin, Frohlich, Loechel, Zolkiewska, Ishiguro, Kojima, Liu, Langford, Sanderson, Brakebusch, Fassler & Wewer (2000) J. Cell Biol. 149, 1143-55 |
| Syndecan-4 | D | ADAMTS1 | Rodriguez-Mazaneque, Carpizo, Plaza-Calonge, Torres-Collado, Thai, Simons, Horowitz & Iruela-Arispe (2009) Int. J. Biochem. Cell. Biol. 41, 800-810 |
| Syndecan-4 | I | Arf6 | Brooks, Williamson, Bass (2012) Small GTPases |
| Syndecan-4 | D | β1-integrin | Whiteford & Couchman (2006) J. Biol. Chem. 281, 32156-63 |
| Syndecan-4 | D | Calcineurin | Finsen, Lunde, Sjaastad, Ostil, Lyngra, Jarstadmarken, Hasic, Nygard, Wilcox-Adelman, Goetinck, Lyberg, Skrbic, Florholmen, Tonnessen, Louch, Djurovic, Carlson & Christensen (2011) Plos One, 6, e28302 |
| Syndecan-4 | D | CASK | Hsueh, Yang, Kharazia, Naisbitt, Cohen, Weinberg & Sheng (1998) J. Cell Biol. 142, 139-151 |
| Syndecan-4 | I | Clathrin | Ohkawara, Glinka & Niehrs (2011) Dev. Cell 20, 303-14 |
| Syndecan-4 | D | Collagen | Vuoriluoto, Hognas, Meller, Lehti & Ivaska (2011) Matrix Biol. 30, 207-17 |
| Syndecan-4 | I | Cortactin | Kinnunen, Kaksonen, Saarinen, Kalkkinen, Peng & Rauvala (1998) J. Biol. Chem. 273, 10702-8 |
| Syndecan-4 | D | CXCL12 | Brule, Charnaux, Sutton, Ledoux, Chagneau, Saffar & Gattegno (2006) Glycobiology 16, 488-501 |
| Syndecan-4 | D | Dishevelled | Munoz, Moreno, Oliva, Orbenes & Larrain (2006) Nat. Cell Biol. 8, 492-500 |
| Syndecan-4 | D | Dynamin II | Yoo, Jeong, Cho, Oh & Han (2005) Biochem. Biophys. Res. Comm. 328 424-31 |
| Syndecan-4 | D | Ezrin | Granes, Berndt, Roy, Mangeat, Reina & Vilaro (2003) FEBS Lett. 547, 212-6 |
| Syndecan-4 | I | FAK | Wilcox-Adelman, Denhez & Goetinck (2002) J. Biol. Chem. 277, 32970-7 |
| Syndecan-4 | D | FGF2 | Tkachenko, Lutgens, Stan & Simons (2004) J. Cell Sci. 117, 3189-99 |
| Syndecan-4 | D | Fibronectin | Woods, Couchman, Johansson & Hook (1986) EMBO J. 5, 665-70 |
| Syndecan-4 | D | Frizzled | Munoz, Moreno, Oliva, Orbenes & Larrain (2006) Nat. Cell Biol. 8, 492-500 |
| Syndecan-4 | I | Fyn | Kinnunen, Kaksonen, Saarinen, Kalkkinen, Peng & Rauvala (1998) J. Biol. Chem. 273, 10702-8 |
| Syndecan-4 | I | K-Ras | Vuoriluoto, Hognas, Meller, Lehti & Ivaska (2011) Matrix Biol. 30, 207-17 |
| Syndecan-4 | D | Laminin | Carulli, Beck, Dayan, Boulesteix, Lortat-Jacob & Rouselle (2012) J. Biol. Chem. |
| Syndecan-4 | D | MMP9 | Brule, Charnaux, Sutton, Ledoux, Chagneau, Saffar & Gattegno (2006) Glycobiology 16, 488-501 |
| Syndecan-4 | D | NF1 | Hsueh, Roberts, Volta, Sheng & Roberts (2001) J. Neuroscience 21, 3764-70 |
| Syndecan-4 | D | PI(4,5)P2 | Koo, Jung, Shin, Han, Mortier, Zimmermann, Whiteford, Couchman, Oh & Lee (2006) J. Mol. Biol. 355, 651-63 |
| Syndecan-4 | D | PKCa | Koo, Jung, Shin, Han, Mortier, Zimmermann, Whiteford, Couchman, Oh & Lee (2006) J. Mol. Biol. 355, 651-63 |
| Syndecan-4 | P | PKCd | Murakami, Horowitz, Tang, Ware & Simons (2002) J. Biol. Chem. 277, 20367-71 |
| Syndecan-4 | D | Rspo3 | Ohkawara, Glinka & Niehrs (2011) Dev. Cell 20, 303-14 |
| Syndecan-4 | I | Src | Kinnunen, Kaksonen, Saarinen, Kalkkinen, Peng & Rauvala (1998) J. Biol. Chem. 273, 10702-8 |
| Syndecan-4 | D | Synbindin | Ethell, Hagihara, Miura, Irie & Yamaguchi (2000) J. Cell Biol. 151, 53-67 |
| Syndecan-4 | D | Syndesmos | Denhez, Wilcox-Adelman, Baciu, Saoncella, Lee, French, Neveu & Goetinck (2002) J. Biol. Chem. 277, 12270-4 |
| Syndecan-4 | D | Synectin | Elfenbein, Rhodes, Meller, Schwartz, Matsuda & Simons (2009) J. Cell Biol. 186, 75-83 |
| Syndecan-4 | D | Syntenin | Zimmermann, Zhang, Degeest, Mortier, Leenaerts, Coomans, Schulz, N'Kuli, Courtoy & David (2005) Dev. Cell 9, 377-88 |
| Syndecan-4 | D | TG2 | Scarpellini, Germack, Lortat-Jacob, Muramatsu, Billet, Johnson & Verderio (2009) J. Biol. Chem. 284, 18411-23 |
| Syndecan-4 | D | Tiam1 | Shepherd, Hard, Murray, Pei & Fuentes (2011) Biochemistry 50, 1296-308 |
| Syndesmos | D | Hic5 | Denhez, Wilcox-Adelman, Baciu, Saoncella, Lee, French, Neveu & Goetinck (2002) J. Biol. Chem. 277, 12270-4 |
| Syndesmos | D | Paxillin | Denhez, Wilcox-Adelman, Baciu, Saoncella, Lee, French, Neveu & Goetinck (2002) J. Biol. Chem. 277, 12270-4 |
| Synectin | D | Myosin VI | Valdembri, Caswell, Anderson, Schwarz, Konig, Astania, Caccavari, Norman, Humphries, Bussolino & Serini (2009) 7, e1000025 |
| Syntenin | I | Arf6 | Zimmermann, Zhang, Degeest, Mortier, Leenaerts, Coomans, Schulz, N'Kuli, Courtoy & David (2005) Dev. Cell 9, 377-88 |
| Syntenin | D | PI(4,5)P2 | Zimmermann, Zhang, Degeest, Mortier, Leenaerts, Coomans, Schulz, N'Kuli, Courtoy & David (2005) Dev. Cell 9, 377-88 |
| Syntenin | D | PTPη | Chung, Cruz & Ariizumi (2011) Eur. J. Immunol. 41, 1794-9 |
| Talin | D | Actin | Hemmings, Rees, Ohanian, Bolton, Gilmore, Patel, Priddle, Trevithick, Hynes & Critchley (1996) J. Cell Sci. 109, 2715-26 |
| Talin | D | FAK | Chen, Appeddu, Parsons, Hildebrand, Schaller & Guan (1995) J. Biol. Chem. 270, 16995-9 |
| Tiam1 | D | IRSp53 | Harmon, Campbell & Ratner (2010) PLoS Patogens 6, e1000956 |
| Tiam1 | D | Rac1 | Worthylake, Rossman & Sondek (2000) Nature 408, 682-8 |
| Vinculin | D | Actin | Johnson & Craig (1995) Nature 373, 261-4 |
| Vinculin | D | Paxillin | Wood, Turner, Jackson & Critchley (1994) J. Cell Sci. 107, 709-17 |
| Vinculin | D | Talin | Gilmore, Wood, Ohanian, Jackson, Patel, Rees, Hynes & Ctritchley (1993) J. Cell Biol. 122, 337-47 |
| WAVE2 | D | Arp2/3 | Takenawa & Suetsugu (2007) Nat.Rev.Mol.Cell Biol. 8, 37-48 |
| WAVE2 | D | IRSp53 | Takenawa & Suetsugu (2007) Nat.Rev.Mol.Cell Biol. 8, 37-48 |
| Wnt | D | Frizzled | Munoz, Moreno, Oliva, Orbenes & Larrain (2006) Nat. Cell Biol. 8, 492-500 |

**Table S1. Literature-curated syndecan-4 interactome.** References for each of the interactions on the interactome. D = direct interaction, I = indirect, P = phosphorylation.
