# Supplementary material for: Syndecan and integrin interactomes: large complexes in small spaces
Source: Curr Opin Struct Biol. 2012 Oct;22(5):583–90. doi: 10.1016/j.sbi.2012.07.003 (PMC3712168; doi:10.1016/j.sbi.2012.07.003)
Supplement: Supplementary file 2 [file mmc2.docx]

| **Partner A** | **Type** | **Partner B** | **Reference** |
| --- | --- | --- | --- |
| α6β4-integrin | D | ErbB2 | Wang, Leavitt, Ramaswamy & Rapraeger (2010) J. Biol. Chem. 285, 13569-79 |
| α6β4-integrin | P | Fyn | Wang, Leavitt, Ramaswamy & Rapraeger (2010) J. Biol. Chem. 285, 13569-79 |
| Actin | D | WAVE2 | Suetsugu, Miki, Yamaguchi, Obinata & Takenawa (2001) J. Cell Sci. 114, 4533-42 |
| Arp2/3 | D | Actin | Blanchoin, Amann, Higgs, Marchand, Kaiser & Pollard (2000) Nature 404, 1007-11 |
| Arp2/3 | D | Cortactin | Uruno, Liu, Zhang, Fan, Egile, Li, Mueller & Zhan (2001) Nature Cell Biol. 3, 259-66 |
| αvβ3-integrin | D | Talin | Beauvais & Rapraeger (2010) J. Cell Sci. 123, 3796-807 |
| αvβ5-integrin | D | Talin | Beauvais & Rapraeger (2010) J. Cell Sci. 123, 3796-807 |
| Calmodulin | D | K-Ras | Villalonga, Lopez-Alcala, Bosch, Chiloeches, Rocamora, Gil, Marais, Marshall, Bachs & Agell (2001) Mol. Cell Biol. 21, 7345-54 |
| CASK | D | Calmodulin | Borg, Lopez-Figueroa, Taddeo-Borg, Kroon, Turner, Watson & Margolis (1999) 19, 1307-16 |
| CASK | D | MINT1 | Borg, Lopez-Figueroa, Taddeo-Borg, Kroon, Turner, Watson & Margolis (1999) 19, 1307-16 |
| CASK | D | Protein 4.1 | Cohen, Woods, Maratia, Walther, Chishti & Anderson (1998) J. Cell Biol. 13, 129-38 |
| Collagen | D | α2β1-integrin | Vuoriluoto, Hognas, Meller, Lehti & Ivaska (2011) Matrix Biol. 30, 207-17 |
| Cortactin | D | Actin | Uruno, Liu, Zhang, Fan, Egile, Li, Mueller & Zhan (2001) Nature Cell Biol. 3, 259-66 |
| CSK | P | Src | Cao, Courchesne & Mastick (2002) J. Biol. Chem. 277, 8771-4 |
| CXCL12 | I | MMP9 | Brule, Charnaux, Sutton, Ledoux, Chagneau, Saffar & Gattegno (2006) Glycobiology 16, 488-501 |
| EphrinB2 | D | EphB4 | Yuan, Hong, Chen, Tsai & Lin (2004) Blood 104, 1025-33 |
| Ezrin | D | Actin | Algrain, Turunen, Vaheri, Louvard & Arpin (1993) J. Cell Biol. 120, 129-139 |
| Fascin | D | Actin | Adams, Clelland, Collett, Matsumura, Yamashiro & Zhang (1999) Mol. Biol. Cell 10, 4177–90 |
| FGF2 | D | FGFR | Filla, Dam & Rapraeger (1998) J. Cell Physiol. 174, 310-21 |
| IGF1R | D | αvβ3-integrin | Beauvais & Rapraeger (2010) J. Cell Sci. 123, 3796-807 |
| IGF1R | D | αvβ5-integrin | Beauvais & Rapraeger (2010) J. Cell Sci. 123, 3796-807 |
| IRSp53 | D | Actin | Takenawa & Suetsugu (2007) Nat. Rev. Mol.Cell Biol. 8, 37-48 |
| IRSp53 | D | WAVE2 | Takenawa & Suetsugu (2007) Nat. Rev. Mol.Cell Biol. 8, 37-48 |
| K-Ras | I | α2β1-integrin | Vuoriluoto, Hognas, Meller, Lehti & Ivaska (2011) Matrix Biol. 30, 207-17 |
| K-Ras | I | MT1-MMP | Vuoriluoto, Hognas, Meller, Lehti & Ivaska (2011) Matrix Biol. 30, 207-17 |
| Laminin | D | α2β1-integrin | Hozumi, Suzuki, Nielsen, Nomizu & Yamada (2006) J. Biol. Chem. 281, 32929-40 |
| Laminin | D | α6β4-integrin | Wang, Leavitt, Ramaswamy & Rapraeger (2010) J. Biol. Chem. 285, 13569-79 |
| MT1-MMP | D | Collagen | Vuoriluoto, Hognas, Meller, Lehti & Ivaska (2011) Matrix Biol. 30, 207-17 |
| NF1 | D | CASK | Hsueh, Roberts, Volta, Sheng & Roberts (2001) J. Neuroscience 21, 3764-70 |
| Paxillin | D | CSK | Turner (2000) Nat. Cell Biol. 2, E231-6 |
| PKA | P | CASK | Huang, Chang & Hsueh (2010) J. Neurochem. 112, 1562-73 |
| Protein 4.1 | D | Actin | Djinovic-Carugo, Gautel, Ylanne & Young (2002) 513, 119-23 |
| Rab5 | I | β1-integrin | Pellinen, Arjonen, Vuoriluoto, Kallio, Fransen & Ivaska (2006) J. Cell Biol. 173, 767-80 |
| Rac1 | D | IRSp53 | Takenawa & Suetsugu (2007) Nat. Rev. Mol.Cell Biol. 8, 37-48 |
| Src | P | Cortactin | Kinnunen, Kaksonen, Saarinen, Kalkkinen, Peng & Rauvala (1998) J. Biol. Chem. 273, 10702-8 |
| Src | P | Paxillin | Turner (2000) Nat. Cell Biol. 2, E231-6 |
| Syndecan-1 | D | α6β4-integrin | Wang, Leavitt, Ramaswamy & Rapraeger (2010) J. Biol. Chem. 285, 13569-79 |
| Syndecan-1 | D | ADAMTS4 | Gao, Plaas, Thompson, Jin, Zuo & Sandy (2004) J. Biol. Chem. 279, 10042-51 |
| Syndecan-1 | I | Arf6 | Zimmermann, Zhang, Degeest, Mortier, Leenaerts, Coomans, Schulz, N'Kuli, Courtoy & David (2005) Dev. Cell 9, 377-88 |
| Syndecan-1 | I | αvβ3-integrin | Beauvais, Ell, McWhorter & Rapraeger (2009) J. Exp. Med. 206, 691-705 |
| Syndecan-1 | I | αvβ5-integrin | Beauvais, Ell, McWhorter & Rapraeger (2009) J. Exp. Med. 206, 691-705 |
| Syndecan-1 | D | β1-integrin | Hayashida, Stahl & Park (2008) J. Biol. Chem. 283, 35435-44 |
| Syndecan-1 | D | CASK | Hsueh, Yang, Kharazia, Naisbitt, Cohen, Weinberg & Sheng (1998) J. Cell Biol. 142, 139-151 |
| Syndecan-1 | D | Collagen | Vuoriluoto, Hognas, Meller, Lehti & Ivaska (2011) Matrix Biol. 30, 207-17 |
| Syndecan-1 | I | Cortactin | Kinnunen, Kaksonen, Saarinen, Kalkkinen, Peng & Rauvala (1998) J. Biol. Chem. 273, 10702-8 |
| Syndecan-1 | D | CXCL12 | Brule, Charnaux, Sutton, Ledoux, Chagneau, Saffar & Gattegno (2006) Glycobiology 16, 488-501 |
| Syndecan-1 | D | EphB4 | Yuan, Hong, Chen, Tsai & Lin (2004) Blood 104, 1025-33 |
| Syndecan-1 | D | Ezrin | Granes, Berndt, Roy, Mangeat, Reina & Vilaro |
| Syndecan-1 | I | Fascin | Chakravarti, Sapountzi & Adams (2005) Mol. Biol. Cell 16, 3678-91 |
| Syndecan-1 | D | FGF2 | Filla, Dam & Rapraeger (1998) J. Cell Physiol. 174, 310-21 |
| Syndecan-1 | I | Fyn | Kinnunen, Kaksonen, Saarinen, Kalkkinen, Peng & Rauvala (1998) J. Biol. Chem. 273, 10702-8 |
| Syndecan-1 | D | IGF1R | Beauvais & Rapraeger (2010) J. Cell Sci. 123, 3796-807 |
| Syndecan-1 | I | K-Ras | Vuoriluoto, Hognas, Meller, Lehti & Ivaska (2011) Matrix Biol. 30, 207-17 |
| Syndecan-1 | D | Laminin | Carulli, Beck, Dayan, Boulesteix, Lortat-Jacob & Rouselle (2012) J. Biol. Chem. |
| Syndecan-1 | D | MMP7 | Li, Park, Wilson & Parks (2002) Cell 111, 635-46 |
| Syndecan-1 | D | MMP9 | Brule, Charnaux, Sutton, Ledoux, Chagneau, Saffar & Gattegno (2006) Glycobiology 16, 488-501 |
| Syndecan-1 | D | MT1-MMP | Endo, Takino, Miyamori, Kinsen, Yoshizaki, Furukawa & Sato (2003) J. Biol. Chem. 278, 40764-70 |
| Syndecan-1 | D | NF1 | Hsueh, Roberts, Volta, Sheng & Roberts (2001) J. Neuroscience 21, 3764-70 |
| Syndecan-1 | P | PKA | Hayashida, Johnston, Goldberger & Park (2006) J. Biol. Chem. 281, 24365-74 |
| Syndecan-1 | D | Rab5 | Hayashida, Stahl & Park (2008) J. Biol. Chem. 283, 35435-44 |
| Syndecan-1 | I | Src | Kinnunen, Kaksonen, Saarinen, Kalkkinen, Peng & Rauvala (1998) J. Biol. Chem. 273, 10702-8 |
| Syndecan-1 | D | Synbindin | Ethell, Hagihara, Miura, Irie & Yamaguchi (2000) J. Cell Biol. 151, 53-67 |
| Syndecan-1 | D | Syntenin | Zimmermann, Zhang, Degeest, Mortier, Leenaerts, Coomans, Schulz, N'Kuli, Courtoy & David (2005) Dev. Cell 9, 377-88 |
| Syndecan-1 | D | Thrombospondin-1 | Chakravarti, Sapountzi & Adams (2005) Mol. Biol. Cell 16, 3678-91 |
| Syndecan-1 | D | Tiam1 | Shepherd, Klaus, Liu, Ramaswamy & DeMali (2010) J. Mol. Biol. 398, 730-46 |
| Syndecan-2 | D | CASK | Cohen, Woods, Maratia, Walther, Chishti & Anderson (1998) J. Cell Biol. 13, 129-38 |
| Syndecan-2 | D | Ezrin | Granes, Berndt, Roy, Mangeat, Reina & Vilaro (2003) FEBS Lett. 547, 212-6 |
| Syndecan-2 | D | NF1 | Hsueh, Roberts, Volta, Sheng & Roberts (2001) J. Neuroscience 21, 3764-70 |
| Syndecan-2 | D | Synbindin | Ethell, Hagihara, Miura, Irie & Yamaguchi (2000) J. Cell Biol. 151, 53-67 |
| Syntenin | I | Arf6 | Zimmermann, Zhang, Degeest, Mortier, Leenaerts, Coomans, Schulz, N'Kuli, Courtoy & David (2005) Dev. Cell 9, 377-88 |
| Syntenin | D | EphrinB2 | McClelland, Seffler-Collins, Kayser & Dalva (2009) Proc. Nat. Acad. Sci. USA 106, 20487-92 |
| Syntenin | D | PTPη | Chung, Cruz & Ariizumi (2011) Eur. J. Immunol. 41, 1794-9 |
| Talin | D | Actin | Hemmings, Rees, Ohanian, Bolton, Gilmore, Patel, Priddle, Trevithick, Hynes & Critchley (1996) J. Cell Sci. 109, 2715-26 |
| Tiam1 | D | IRSp53 | Harmon, Campbell & Ratner (2010) PLoS Patogens 6, e1000956 |
| Tiam1 | D | Rac1 | Worthylake, Rossman & Sondek (2000) Nature 408, 682-8 |
| Vinculin | D | Actin | Johnson & Craig (1995) Nature 373, 261-4 |
| Vinculin | D | Paxillin | Wood, Turner, Jackson & Critchley (1994) J. Cell Sci. 107, 709-17 |
| Vinculin | D | Talin | Gilmore, Wood, Ohanian, Jackson, Patel, Rees, Hynes & Ctritchley (1993) J. Cell Biol. 122, 337-47 |
| WAVE2 | D | Arp2/3 | Takenawa & Suetsugu (2007) Nat. Rev. Mol.Cell Biol. 8, 37-48 |

**Table S2. Literature-curated syndecan-1 interactome.** References for each of the interactions on the interactome. D = direct interaction, I = indirect, P = phosphorylation.
