# Supplementary material for: Syndecan and integrin interactomes: large complexes in small spaces
Source: Curr Opin Struct Biol. 2012 Oct;22(5):583–90. doi: 10.1016/j.sbi.2012.07.003 (PMC3712168; doi:10.1016/j.sbi.2012.07.003)
Supplement: Supplementary file 3 [file mmc3.docx]

|  | **Residue** | **Kinase / phosphatase** | **Permits binding of** | **Notes** | **Reference** |
| --- | --- | --- | --- | --- | --- |
| Syndecan-1 | S-285 | PKA | TGFβ | The authors investigated the mouse variant of syndecan-1 and highlights S-286 as the target residue. This corresponds to human S-285 | Hayashida et al. 2006 |
|  | Y-286?  Y-299?  Y-309? | Src | Unknown protein tyrosine kinases | Src activity may promote cleavage of ectodomain although there is conflicting evidence for this (see references). | For: Ott and Rapraeger 1998; Reiland et al. 1996; Fitzgerald et al. 2000.  Against: Hayashida et al. 2008 |
|  | Y-309 | Unknown tyrosine phosphatase | De-phosphorylated state allows binding of syntenin | This residue may be constitutively phosphorylated to regulate binding of syntenin. | Sulka et al. 2009 |
| Syndecan-2 | S-187 | PKC | ? | Residue numbers noted in reference (Oh et al. 1997) refer to rat syndecan-2 protein. Rat and human cytoplasmic domains are identical. | Oh et al. 1997  Prasthofer et al. 1995 |
|  | S-188 | PKC | ? | Residue numbers noted in reference (Oh et al. 1997) refer to rat syndecan-2 protein. Rat and human cytoplasmic domains are identical. | Oh et al. 1997 |
|  | Y-179 | EphB2 | Syndecan clustering | Phosphorylation by EphB2 at this site may regulate clustering of syndecan-2 together with phosphorylation of Y-191 | Ethell et al. 2001 |
|  | Y-191 | EphB2 | Syndecan clustering | Phosphorylation by EphB2 at this site may regulate clustering of syndecan-2 together with phosphorylation of Y-179 | Ethell et al. 2001 |
| Syndecan-3 | Y-419 | Src | Cortactin?  Tubulin? | Both cortactin and tubulin, together with Src co-purify from brain lysates | Kinnunan et al. 1998 |
|  | Y-419?  Y-431?  Y-441? | PKCδ?  Src?  Unknown tyrosine kinase | ? | The authors also suggest Y-409 may be phosphorylated in addition to the 3 cytoplasmic domain tyrosine residues | Asundi and Carey 1997 |
| Syndecan-4 | S-179 | PKCδ  Tiam1? (see patent by Szilak et al. 2011)  Calcineurin A (see Finsen et al. 2011) | Syndecan-4/PIP_2_/PKCα complex | Phosphorylation at this residue may influence phosphorylation by Src at site 180. S-179 phosphorylation also promotes syndecan-4 ectodomain shedding and correct cytokinesis. There is also evidence to support α-actinin binding the V domain upon S-179 phosphorylation. Koo and coworkers note that phosphorylation at this site causes conformational change in C1 and C2 domains thus altering the exposure of Y-180 and Y-197. PKCδ-mediated phosphorylation of S-179 causes a transient PKCα activation following matrix engagement. Calcineurin subunit A binding is also reduced when S-179 is de phosphorylated. | Couchman et al 2010; Keller-Pinter et al. 2010; Koo et al 2006; Finsen et al. 2011 |

**Table S3. Current evidence for tyrosine and serine phosphorylation within the syndecan family cytoplasmic domain**. Note that all residue numbers referred to in this table are for the human form of the syndecan protein regardless of whether the study was performed in another species.
